# The Doctor's Christmas Box

**Published:** 1892-01-09

**Authors:** 


### The Hospital

A Weekly Journal of

Science, Medicine, Nursing, and Philanthropy.

*For Hospitals, Asylums, Infirmaries, Dispensaries, Medical Practitioners, Students, Nurses, and the Charitable Public.*

Vol. XI.—No. 276.] [Jan. 9, 1892.

### THE DOCTOR'S CHRISTMAS BOX.

It is the custom of the doctor's richer patients to send him at Christmas time some more or less substantial token of their gratitude and good-will. To many people the annual demands of the postman and the policeman, the dustman and the turncock, the greengrocer's assistant, the butcher's shiny-haired retainer *et hoc genus omne*, are rapidly becoming a burden too grievous to be borne. But the doctor's Christmas-box is quite another thing. He does not present himself in the area, or even at the front door, to ask for it; he does not so much as anticipate it. He is pleased when it comes, because he knows his patients are pleased with him when they send it; and that constitutes its real value to him. Moreover, he does not encourage his less wealthy patients to send him any Christmas-box at all: he would rather they kept it at home. But a dozen bottles of rich and mature port from a millionaire is a mere bagatelle; and the doctor can accept it without any loss of dignity, and with the feeling that his careful and kindly services have been appreciated, and that he still retains the confidence of his patient.

It is not, however, of this kind of Christmas-box that we wish to speak, but of one that is to the family doctor a matter of real, and sometimes of urgent importance. At Christmas time the family physician sends in his "little bill." As a rule it is really a "little bill" when it is compared with all the anxious work which it represents. The average medical man of the present day charges very small fees. In Scotland one and sixpence is often considered sufficient remuneration for a visit to a small farmer or a respectable tradesman. In England half-a-crown is paid by the same class of people; but in the latter case a bottle of physic is included in the charge for the visit. Three and sixpence is held to be a respectable fee in a middle-class London suburb; and a man who has reached the position of charging five shillings a visit all round without physic, is looked upon by many of his less fortunate brethren as a very Paladin of professional dignity, and a Crœsus for wealth.

There is something pathetically ludicrous about this state of things. The thoroughly successful family doctor is, as a rule, a man of great discretion, high character, scientific culture, and trained practical capacity. He is the kind of man who, if he had gone into a wholesale or even a retail business and spent as much money and diligent industry upon it at the outset of his career as he spent in preparing himself for entrance into the medical profession, would have counted his gains by a good many thousands a year before he had reached the sober age of fifty. But all his efforts will not enable him to count his actual net gains by much more than one thousand a year at the age of fifty as a medical man; fortunate, indeed, is he if they are nearly so much; and in order to secure even so much he is compelled to work almost night and day, and to deprive himself of needed daily recreation and of practically every joy in life. The high-class general practitioner who, after nearly half a century's hard scientific work and unparalleled devotion to duty is worth ten thousand pounds at the age of sixty-five, is an extremely rare person to meet with. The earnest, kindly, and genial doctors who are not worth so much as one thousand pounds at the age of seventy may be numbered, even in this country, by thousands, and almost by tens of thousands.

The beneficed clergyman of the Church of England can generally retain his living, by the help of one or more curates, to the last day of his life; and the ministers of the unendowed churches have their retiring funds for old age, and their sympathetic congregations to make up for each of them a capital sum when they become too feeble for further work. But the family physician knows of no retiring fund for worn out medical men: and whoever heard of a body of patients meeting together and subscribing so much as even a couple of thousands of pounds for the medical man who had attended, perhaps, three generations of families in the same neighbourhood? The writer knows of one minister's widow, who, though not at all in straitened circumstances, when her husband died at the fairly full age of sixty-five, had six thousand pounds presented to her by his friends and admirers. There are many doctors who have deserved as well of their patients as that particular minister had of his congregation; but one would as soon expect a collection of patients to give their doctors six millions as six thousand pounds.

It is generally considered that the obligations of patients to doctors are sufficiently discharged when the annual account rendered by the family medical man is punctually paid. That, we admit, may be so; but that is the very point to which we desire to bring non-professional readers at the present time. The chief of all the reasons for the chronic poverty of many members of the medical profession is that their annual accounts are not punctually paid. Even some of those who send the doctor a dozen of port or a case of champagne at Christmas withhold from him the far more important consideration of the prompt payment of his account. We are justified in stating that the average doctor seldom receives more than four-fifths of his actual earnings. Putting together the work that he voluntarily does for nothing, and the loss which he sustains annually by unpaid accounts, the doctor who earns a thousand a-year does not get more than eight hundred. When to this is added the fact that the eight hundred is paid in the most irregular of all fashions, some of it remaining on the doctor's books for two or three years, it is easy to understand how a man with a nominal income of eight hundred a-year may, after he has paid the cost of running his carriage, his assistant, and his wholesale druggist's bill, be left with a net income on which it is almost impossible to live.

If the doctor who earns a thousand a-year were paid punctually by all his patients, the loss of a fifth of his earnings would be avoided, and the hundred and fifty pounds a-year thus secured would make all the difference between straitened means and assured prosperity. He would be able to save from his income as much as a hundred a-year, and thus to secure for his old age, if not competence, at least a provision against positive want. We can do in these pages what the doctor cannot do for himself without loss of dignity: we can explain the facts of the case as they affect doctors generally; and the facts themselves make the most convincing appeal to every right-minded man who wishes to do as he would be done by. The average doctor would be quite able to provide a modest competence for old age if he could but get what he has earned, at the proper time. But when he finds that the money receipts from a year's anxious toil are barely sufficient to meet current expenses, and that during the costly period of his children's education, he loses ground rather than gains it, who can wonder that he becomes depressed and disheartened, and looks like a white-haired gentleman of sixty when the actual years of his age are under fifty?

The public is not wise to use its professional men so hardly as it does. The lawyer can take care of himself. Few people venture to question his account, and none but the boldest dares to delay the payment of it. The lawyer, too, has many ways of making money which are quite closed against the doctor. He knows the best bargains that are going in all the markets of the world, and practically always falls upon his feet if he be a man of any worth at all. But the case is very different with the doctor, as it is also with the clergyman and the schoolmaster. They are not men of business; they depend upon their earnings and savings alone; and it is of the utmost importance to them that their earnings be paid promptly and paid in full.

The martyrdom of the medical profession means in the long run the injury of its employers. Everybody knows the difference between a London cab-horse and a prime-conditioned hunter in the country. There is as much difference between the brain-power of a hard-driven, ill-paid doctor, and a doctor who is flourishing and free from care, as there is between the paces of the sorry cab-horse and those of the splendid hunter. The poor man who is constantly harassed by the practical impossibility of making both ends meet cannot devote heart and mind and soul to the physical ills of his patient. His own mental ills are so paralysing to his mind that he sometimes hardly knows what he is doing. If he were better and more punctually paid he would be better fed; he would get more holidays; he could buy more books; he would find time to meet his professional brethren at their medical societies and scientific gatherings. He would be in every respect a new and more capable man. For the doctors' sake, therefore, and for the patients' sake quite as much or even more, we counsel every patient to send to his medical man the only Christmas box he ever desires or asks for—the punctual payment of what he has laboriously earned.

